# Tissue Microarray Technology for Molecular Applications: Investigation of Cross-Contamination between Tissue Samples Obtained from the Same Punching Device

**DOI:** 10.3390/microarrays4020188

**Published:** 2015-04-02

**Authors:** Erik Vassella, José A. Galván, Inti Zlobec

**Affiliations:** Translational Research Unit (TRU), Institute of Pathology, University of Bern, Murtenstrasse 31, Room L313, CH-3010 Bern, Switzerland; E-Mails: erik.vassella@pathology.unibe.ch (E.V.); jose.galvan@pathology.unibe.ch (J.A.G.)

**Keywords:** tissue microarray, biomarker, digital pathology

## Abstract

Background: Tissue microarray (TMA) technology allows rapid visualization of molecular markers by immunohistochemistry and *in situ* hybridization. In addition, TMA instrumentation has the potential to assist in other applications: punches taken from donor blocks can be placed directly into tubes and used for nucleic acid analysis by PCR approaches. However, the question of possible cross-contamination between samples punched with the same device has frequently been raised but never addressed. Methods: Two experiments were performed. (1) A block from *mycobacterium tuberculosis* (TB) positivetissue and a second from an uninfected patient were aligned side-by-side in an automated tissue microarrayer. Four 0.6 mm punches were cored from each sample and placed inside their corresponding tube. Between coring of each donor block, a mechanical cleaning step was performed by insertion of the puncher into a paraffin block. This sequence of coring and cleaning was repeated three times, alternating between positive and negative blocks. A fragment from the 6110 insertion sequence specific for *mycobacterium tuberculosis* was analyzed; (2) Four 0.6 mm punches were cored from three *KRAS* mutated colorectal cancer blocks, alternating with three different wild-type tissues using the same TMA instrument (sequence of coring: G12D, WT, G12V, WT, G13D and WT). Mechanical cleaning of the device between each donor block was made. Mutation analysis by pyrosequencing was carried out. This sequence of coring was repeated manually without any cleaning step between blocks. Results/Discussion: In both analyses, all alternating samples showed the expected result (samples 1, 3 and 5: positive or mutated, samples 2, 4 and 6: negative or wild-type). Similar results were obtained without cleaning step. These findings suggest that no cross-contamination of tissue samples occurs when donor blocks are punched using the same device, however a cleaning step is nonetheless recommended. Our result supports the use of TMA technology as an accessory to PCR applications.

## 1. Introduction

Tissue microarrays (TMAs) play an important role in translational and clinical studies [1]. By concentrating hundreds of small tissue cores typically of 0.6 mm in diameter onto a single paraffin block, dozens of biomarkers can be studied on a large number of patients while at the same time sparing costs, resources and tissues [2,3,4]. TMAs are used to study protein markers by immunohistochemistry and to investigate DNA aberrations, mRNA and miRNA expression using various *in situ hybridization* techniques [5,6,7]. Together with clinically annotated and appropriately powered sample cohorts, TMAs are a powerful tool not only for biomarker screening but also for prognostic and predictive modeling of disease outcome. In addition, TMAs allow that the same experimental conditions be applied to hundreds of samples simultaneously [8].

**Figure 1:**
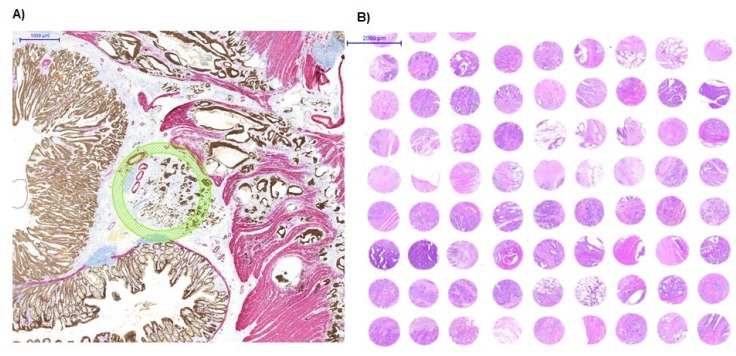
(**A**) Digital annotation of a scanned slide after double-immunohistochemistry staining with pan-cytokeratin and caldesmon; (**B**) Hematoxylin and Eosin staining of a tissue microarray (TMA) constructed from annotated regions of histological interest.

Recent advances in TMA technology rely on digital pathology and automated tissue microarraying. One approach is to use a slide scanner to digitally visualize stained tissue sections, which can then be annotated using a TMA tool of various sizes (Figure 1). These annotated slides are matched to their corresponding donor blocks, which are precisely punched out at the desired locations and transferred into recipient blocks, automatically. At our institute, this approach of combining modern TMA technology with histopathological expertise and biostatistics is referred to as next-generation TMA (ngTMA) [9].

Clearly, TMA instrumentation could also be advantageous for applications other than construction of TMAs. In fact, punches taken from donor blocks can be placed directly into tubes and therefore used for molecular analysis using PCR-based approaches.

However, the question of possible cross-contamination across tissue samples using the same punching device, especially one embedded in an automated tissue microarrayer, has frequently been posed but never addressed. This may be of particular concern if nucleic acids are amplified by PCR. Therefore, in this study, we determine the level of contamination transferred between samples after automated punching using a sensitive assay for mycobacteria and a pyrosequencing assay for *KRAS* mutation analysis.

## 2. Experimental Design

### 2.1. Patients

Mycobacteria assay: In a first step, we identified two patients recently diagnosed at the Institute of Pathology, University of Bern. The first was a 21 year-old male with a histological diagnosis of granulomatous lymphadenitis compatible with tuberculosis. A molecular test for *mycobacterium tuberculosis*, *bovis* and *BCG* was made and confirmed the presence of the bacteria. The second was a 67 year-old woman with right-sided colon cancer and no evidence of mycobacterial infection. Formalin-fixed paraffin-embedded tissue blocks from the lymph nodes and cancer, respectively, were retrieved from the tissue archive.

*KRAS* assay: In a second step, we identified six patients also recently diagnosed with metastasizing colorectal cancer diagnosed at the same Institute. All underwent mutation analysis for *KRAS* Exon 2, codons 12 and 13 using pyrosequencing. Three patients were wild-type (WT); one patient had a colorectal cancer with G12D mutation, the second a G12V mutation and the third was mutated in codon 13 (G13D).

### 2.2. Analysis of Alternating Mycobacterium Positive and Negative Tissue Samples

**Figure 2:**
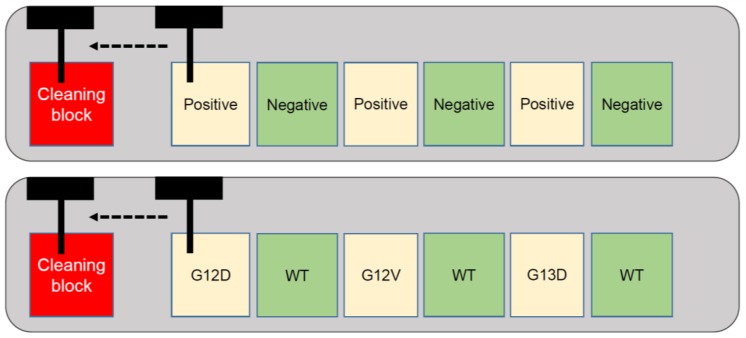
Schematic diagram showing (**above**) the alternating sequence of *mycobacterium*-positive and negative tissue blocks in the arrayer; (**below**) alternating colorectal cancer tissue blocks with known mutational status, followed by mechanical cleaning of the punching device in a paraffin block.

First the mycobacterium-positive and then negative tissue blocks were loaded into an automated tissue microarrayer (TMA Grandmaster, 3D Histech, Budapest, Hungary). Four punches at 0.6 mm in diameter were taken from the positive sample and transferred to a 0.2 mL PCR tube. The punching tool was mechanically cleaned in an empty paraffin block by insertion and removal multiple times (Figure 2). Then, the negative tissue block was cored similarly and tissue punches placed into the respective tube. Again a mechanical cleaning took place. This sequence was repeated three times such that six different tubes containing alternating positive and negative samples were obtained.

DNA was isolated from tissue punches by overnight digestion with proteinase K following purification using a BioRobot EZ1 (Qiagen, Hilden, Germany). The primer pair used for PCR amplification of a fragment from the 6110 insertion sequence specific for *mycobaterium tuberculosis* complex was 5'-CCTGCGAGCGTAGGCGTCGG-3' and 5'-GTTTCTCGTCCAGCGCCGCTTCGG-3'. The forward primer was labeled by FAM. Amplification was performed in 40 cycles of 95 °C for 1 min, 62 °C for 1 min and 72 °C for 1.5 min. The PCR product was analyzed by capillary electrophoresis using a Genetic Analyzer 3500 (Life Technologies, Zug, Switzerland). 5 fg of *mycobacterium tuberculosis* DNA corresponding to 5–10 bacteria was used as a positive control. Human DNA was used as a negative control.

### 2.3. Analysis of Alternating KRAS Mutated and Wild-Type Tissue Samples

In order to compare the level of contamination between a manual punching device without any cleaning step between donor blocks and the automated device with mechanical cleaning step described above, *KRAS* analysis was performed. Using a punching device from a homemade semi-automated tissue microarraying instrument, four punches of 0.6 mm in diameter were cored out from the tumor blocks in the following sequence: G12D, WT, G12V, WT, G13D, and WT. Punches were placed into separate tubes for further analysis. Next, the same tissue blocks were loaded into the automated tissue microarrayer and punched out using a 0.6 mm tool × 4 times according to the same sequence, as above. Between each block, a mechanical cleaning step was performed by insertion of the device into an empty paraffin block several times.

DNA was extracted using standard protocols (QIAamp^®^ DNA FFPE Tissue, Qiagen). PCR was performed using the Pyromark PCR kit (Qiagen) with the following primer sequences for *KRAS* (Microsynth^®^, Balgach, Switzerland): Forward 5'-TAA GGC CTG CTG AAA ATG ACT G-3', Reverse 5'-TTA GCT GTA TCG TCA AGG CAC TCT-3' and Sequencing 5'-CTT GTG GTA GTT GGA GC‑3'. The PCR conditions were as follows: activation step at 95 °C for 15 min, denaturation 30 s at 94 °C, annealing 35 cycles of 30 s at 60 °C, and extension 30 s at 72 °C and final extension 10 min at 72 °C. After PCR, fragment analysis was carried out using a Qiaxcel system (Qiagen). Then, the mutation analysis of *KRAS* (exon 2, codon 12 and 13) was performed using the pyrosequencing method using a PyroMark Q24 (Qiagen). The sequence to analyze was TGNTGRCGTAGGCAAGAGT GCCTTGACGATA. In addition, a control oligo and water control were used in the pyrosequencing as well as appropriate positive and negative of *KRAS* mutation controls.

## 3. Results/Discussion

### 3.1. An Alternate Use of TMA Technology

TMA technology combined with digital pathology can help strengthen biomarker research [10]. Recent advances allow precise histological areas to be digitally annotated onto scanned tissue slides. These annotations are then used to identify specific regions from the corresponding tissue block that should be punched out and transferred to the TMA. Another possible application of this technology is to punch out and transfer tissues directly into tubes, which can then be used for subsequent molecular analysis. However, the possibility for cross-contamination between samples has not yet been addressed. This contamination could arise from tiny amounts of material remaining on the punching device that can be transferred from one tissue sample to the next.

### 3.2. Cross-Contamination Assessed Using Mycobacterium Tuberculosis Assay

**Figure 3:**
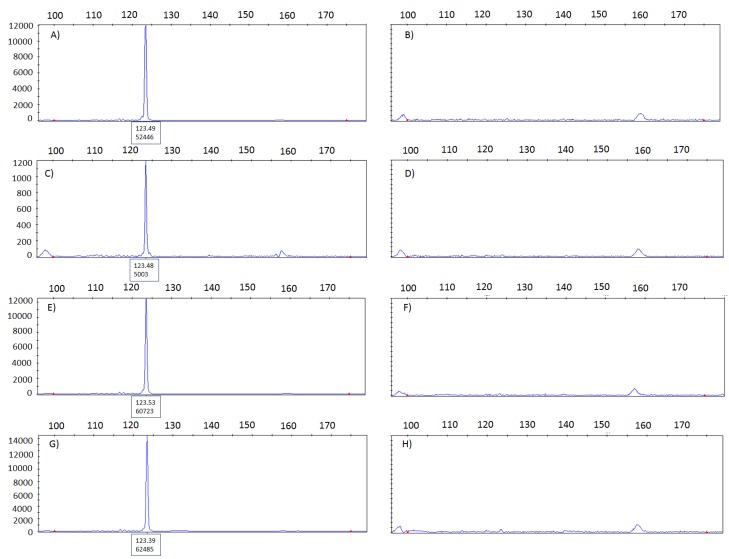
Electropherograms used to evaluate the presence of mycobacterium in donor blocks from a previously confirmed positive and negative tissue sample. (**A**) positive control and (**B**) negative control. Tissue samples were alternated in the tissue arrayer three times leading to the following results: (**C**,**E**,**G**) *mycobacterium tuberculosis* samples and (**D**,**F**,**H**) *mycobacterium tuberculosis* negative samples. No evidence for cross-contamination between samples.

To assess cross-contamination between punches, we alternate *mycobacterium tuberculosis-*positive and negative tissue blocks in an automated tissue arrayer with a mechanical cleaning step in between. Tissue blocks were punched out sequentially. The presence of mycobacterial DNA in the tissue punches was analyzed by PCR using a primer pair specific for IS6110, an element, which is present at multiple copies in the genome of *mycobacterium tuberculosis* complex [11]. This method is proven to be highly sensitive as it allows for the detection of as little as 1–2 mycobacteria. A positive mycobacterium result would be indicated by a peak at 123 bp in the corresponding electropherogram. A fragment of 123 bp was clearly detected in the DNA from punches from *mycobacterium tuberculosis-*positive tissues (Figure 3A,C,E). In contrast, *mycobacterium*-negative tissues punched out in between, were found to be negative (Figure 3B,D,F). Thus, there appears to be no transfer of *mycobacterium tuberculosis* from the positive to the negative case.

### 3.3. Cross-Contamination Assessed Using KRAS Analysis by Pyrosequencing

**Figure 4:**
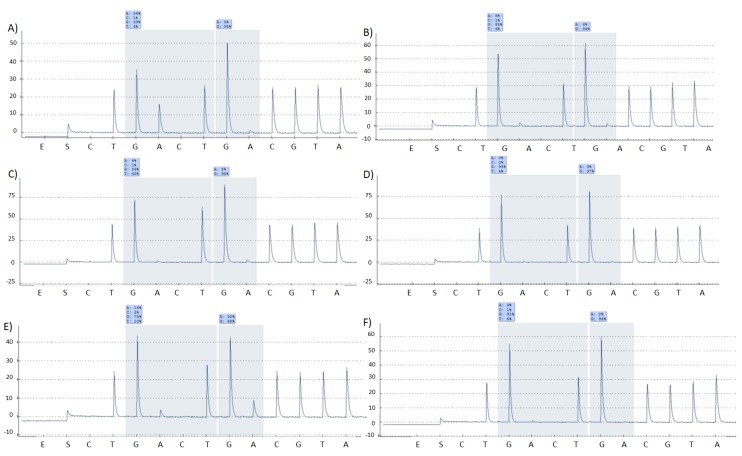
Pyrograms highlighting results from an analysis of KRAS exon 2 codon 12 and 13. (**A**) G12D mutation; (**B**) WT; (**C**) G12V mutation; (**D**) WT; (**E**) G13D mutation; **(F**) WT.

Since the detection of mycobacterium in the negative samples may be influenced by the level of infection, we carried out the analysis of *KRAS* mutational status on colorectal cancers. Using only a manual punching device without cleaning step and alternating between tissue samples, no cross‑contamination could be seen. When analysis of KRAS gene mutation was performed using the automated device with cleaning step, again no cross-contamination of tissue samples was found. All mutated samples carried the same mutations, while all WT samples were clearly WT (Figure 4).

### 3.4. Impact of the Finding

Researchers working with modern TMA technologies have a growing interest in the possible application of TMA instrumentation for purposes other than TMA block construction. Especially, a combined digital pathology/TMA approach is ideal for assessing issues of heterogeneity and allows very precisely annotated histological regions to be transferred either to TMAs or to tubes for storage and/or analysis of nucleic acids. With or without a digital pathology platform, neighboring punches side-by-side for TMA construction and simultaneous molecular analysis is clearly advantageous in the era of molecular pathology.

This study was motivated by the growing concerns over cross-contamination between samples and appears to be the first study to date to address this issue. Using two different PCR methods and alternating between positive/mutated and negative/wild-type samples, our findings underline no evidence of transfer of material from one tissue block to the next.

### 3.5. Conclusion

To conclude, our finding suggests that TMA instrumentation is appropriate for use as an accessory to molecular applications. No cross-contamination appears to occur between samples punched with the same device, although a cleaning step in between donor blocks is still recommended.
